# Barriers and facilitators to tuberculosis service access for patients in Bajhang, Nepal: a qualitative study based on focus groups with patients, screening camp visitors, community volunteers and medical doctors

**DOI:** 10.1186/s41043-025-00788-4

**Published:** 2025-02-21

**Authors:** Emil Malla, Sachin Shahi, Birger Forsberg

**Affiliations:** 1 Karolinska Institute, Stockholm, Sweden; 2 Seti Provincial Hospital, Kailali, Dhangadhi, Nepal

**Keywords:** Tuberculosis, Healthcare access, Barriers and facilitators, Patient, Rural areas, Sudurpaschim Province of Nepal

## Abstract

**Background:**

Efforts to achieve global tuberculosis (TB) targets, especially in lower- and middle-income countries, such as Nepal, require urgent attention. Challenges persist, including TB-related social stigma and catastrophic costs incurred by affected households. Socioeconomically vulnerable individuals, particularly those residing in rural, mountainous and remote areas, face limited access to TB care. To explore the barriers and facilitators to accessing TB care, this study was conducted in Bajhang, one of the most underdeveloped and socioeconomically vulnerable districts in Sudurpaschim Province of far-western Nepal.

**Methods:**

Focus group discussions (FGDs) were held with TB patients, TB camp visitors, community mobilizers and medical doctors.

**Results:**

Thirty-five barriers and 21 facilitators were identified by thematic analysis of the eight FGDs. Key barriers included economic burdens, social stigma leading to concealment and isolation, limited resources in local healthcare and geographical inaccessibility. Notable facilitators included TB volunteer programs, screening camps, economic support, and awareness campaigns.

**Conclusions:**

Prioritizing actions should target early care cascade stages and address individual, community, infrastructure, and health policy levels.

## Background

Improving accessibility to TB care remains a key challenge in ending the global tuberculosis (TB) epidemic, especially in low- and middle-income countries (LMICs), such as Nepal. Despite TB being a preventable and curable disease, 10.6 million people became ill, and 1.6 million people died due to TB worldwide in 2021 [1]. Increased incidence, mortality and multidrug-resistant TB (MDR-TB) cases were observed during the COVID-19 pandemic [1]. Forty-eight percent of people with TB face catastrophic costs, defined by the WHO as “ > 20% of household expenditure or income” [2].

Nepal is a high-burden TB country with an estimated prevalence of 117,000 TB cases and incidence of 69,000 TB cases [3]. TB accounts for one of Nepal’s top ten causes of death [4]. Additionally, the high burden of MDR-TB in Nepal put the country on the WHO watchlist in 2021 [5]. Estimated incidence for all types of TB in Nepal has declined three percent yearly in the last decade, which is faster than the global trend, but the reported numbers of TB cases are considerably uncertain. There is a risk of bias in interpreting reported TB cases in settings of poor access to TB care, as the reported numbers may not adequately reflect the true TB incidence [6]. A higher reduction rate is required to reach the Sustainable Development Goals (SDGs) target by 2035 [3].

TB services are delivered through public and private healthcare facilities at the levels of administrative divisions according to the new federal government system in Nepal, on the local, provincial, and federal level [7]. These levels correspond to primary, secondary and tertiary health facilities respectively and are linked through a referral system. Secondary and tertiary health facilities include provincial and federal hospitals, which are referral points for primary facilities [7]. At the local level, primary health facilities including health posts and female community health volunteers (FCHV) operate as the first point of care for the patient [7].

The FCHV program is a nationwide network of trained volunteers established in the 1980s engaging their local communities with healthcare [8]. The role of FCHVs is to promote healthy behaviors and provide basic health services in their local communities, such as distributing drugs [9]. TB volunteers, with a similar role to FCHVs but with emphasis on promoting TB health services, and TB screening camps are other regional implementations of the National Tuberculosis Program (NTP) [10]. TB screening camps is another line of primary health facilities that the municipality organizes locally a few times a year in selected areas identified as high risk, such as where previous TB cases have been identified. People in the area with suspect TB can come and get tested at the screening camps [10]. Community volunteers and TB camp visitors were identified as key stakeholders in this study because they were deemed to provide valuable information on what barriers and facilitators people face in accessing TB care while also providing different perspectives from patients and caregivers at health facilities.

TB services include both diagnostic and treatment services, but these are not always available from the same health facility. Nationally, while 2 out of 3 health facilities provide TB treatment, only 23% offer any type of diagnostic services for TB. Out of the facilities providing any TB service only 12% had smear microscopy and 13% X-ray. There are around 600 microscopy centers nationally and a growing number of centers operate GeneXpert, which is a rapid molecular diagnostic test for TB and antibiotic sensitivity. [7]. Directly Observed Therapy Short-course (DOTS) is provided as the standard treatment at more than 4000 treatment centers which are operated on a municipality level, and out of which 21 are specialized on DR-TB[3]. Economic support is provided by the current NTP in the form of free testing and free treatment for people with TB and nutritional support for people with MDR-TB. Although access to TB care has improved in Nepal 30% of TB cases [11] and 55% of MDR-TB cases [4] are estimated to be missed by the healthcare system.

The known barriers and facilitators in accessing TB care may be divided into economic, social, psychological, geographical, demographical, disease/treatment-related, and facility-related aspects among others [12]. To identify and treat missing TB cases, more research is needed on the mechanisms behind barriers to access TB care viewed from the many dimensions of access and from the perspective of multiple stakeholders [13]. Access to TB care has previously been researched in Nepal [14–16] and in other high-burden LMICs [12]. However, Bajhang and other hilly districts of the Far-Western region have not been studied. The objective of this study was to explore the barriers to and facilitators of TB diagnosis and treatment in Bajhang as perceived by key stakeholders. Ultimately, this knowledge has a potential to improve the development of more effective strategies for the elimination of TB.

## Methods

### Study design

To explore the barriers to and facilitators of accessing TB care as perceived by key stakeholders in Bajhang, a qualitative study method was designed based on focus group discussion (FGD) [17]. FGDs allow stakeholders to explore topics and generate insights that would otherwise be lost in individual interviews [18]. The use of FGDs was prioritized to gain valuable perspectives and complexity that are more easily raised when the participants discuss issues together, rather than individually.

## Research team

The research team consisted of a Swedish professor, a Swedish-Nepali medical student, a Nepali medical doctor, and a Nepali research assistant.

### Study setting

**Fig. 1:**
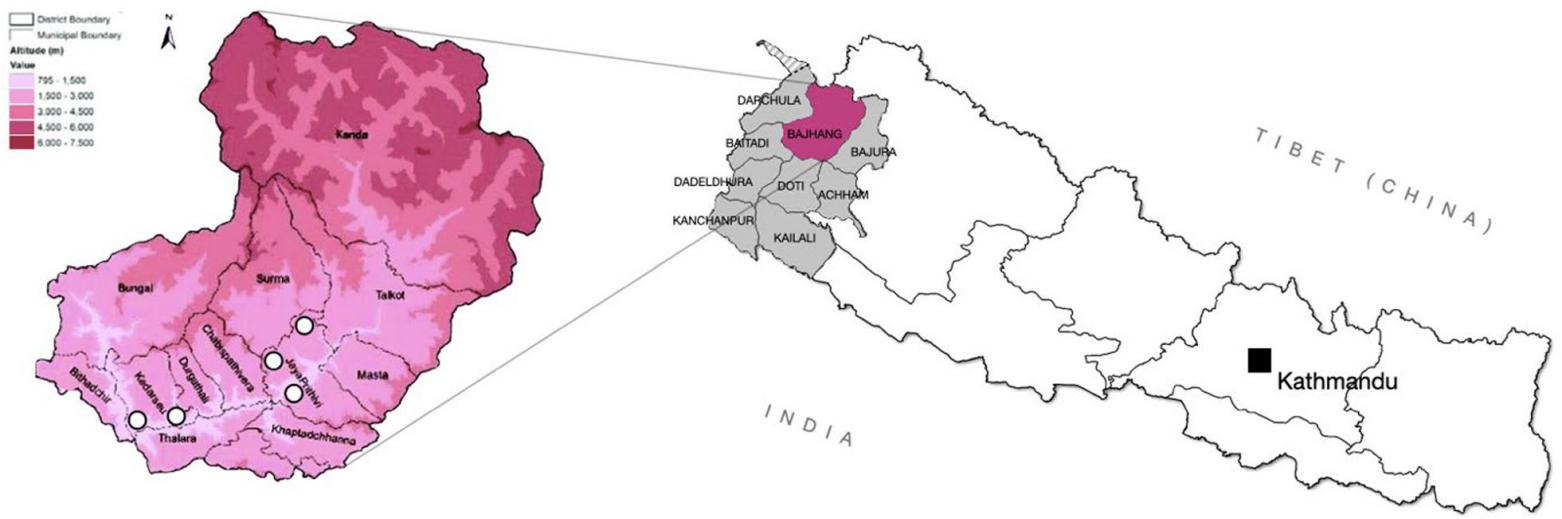
Map of the study setting To the right: map of Nepal with the Far-Western region highlighted in gray and the district of Bajhang highlighted in purple. To the left: Topographic map of Bajhang with the locations Deura, Jhota, Chainpur, Chayala and Siddhamanana where focus group discussions were conducted are marked with white circles. Image was edited under Creative Commons Attribution 4.0 International, CC BY 4.0 [25]

FGDs were held in five locations in Bajhang: in the district hospital in Chainpur, at local health centers in Deura and Jhota, and at TB screening camps held in Siddhamanana and Chayala (Fig. 1).

Bajhang is a hilly district in Far-Western Nepal with a population of 195,159 [19]. It is one of the most remote, socioeconomically vulnerable and underdeveloped districts of Nepal, with a Human Development Index of 0.365 compared to 0.632 in Kathmandu [20]. The case notification rate of TB in Bajhang is 54 per 100,000, compared to the national average of 109 per 100,000 [4]. A total of 58.3% of the population in Bajhang lives more than one hour from their closest health post [21]. The study setting was chosen due to the accessibility to the research team through an established network in the area.

### Key stakeholders

FGDs were held with representatives of four categories of key stakeholders: TB patients, TB camp visitors, medical doctors (MDs) and community mobilizers (CMs), including female community health volunteers (FCHVs) and TB volunteers. The different groups of stakeholders were included to increase reliability of findings. A total of 35 participants were included (Table 1).

**Table 1:** Characteristics and constellations of study participants in focus group discussions

Key stakeholder group	Sex	Age (years)	Place of FGD
Visitors at TB camp	M	–	Siddhamanana
	M	–	
	M	–	
	F	–	
	F	–	
Visitors at TB camp	F	–	Siddhamanana
	F	–	
	F	–	
	F	–	
Visitors at TB camp	M	–	Chayala
	M	–	
	F	–	
	F	–	
Patients with TB	F	20–30	Deura
	F	20–30	
	F	20–30	
	F	30–40	
	M	50–60	
Patients with TB	M	40–50	Jhota
	F	40–50	
	M	30–40	
	F	< 20	
	F	30–40	
Patients with TB	M	30–40	Chainpur
	M	60–70	
Medical doctors	M	20–30	Chainpur
	M	20–30	
	M	30–40	
	M	30–40	
	M	30–40	
Community mobilizers (TB volunteers, FCHVs)	F*	30–40	Chainpur
	F*	20–30	
	M*	20–30	
	F**	50–60	
	F**	30–40	
Total:	All: 35 Male: 16 Female: 19		

^*^TB volunteer, **female community health volunteer. Abbreviations: FCHV = female community health volunteer, FGD = focus group discussion, F = female, M = male, TB = tuberculosis

### Sampling

Convenience sampling [17] was used to recruit participants to the FGDs. Patients with TB were sampled from medical records spanning the past year, provided by District Hospital Shimkhet Bajhang and at DOTS centers in Deura, Jhota and Chainpur. Community mobilisers, medical doctors and TB patients who were currently undergoing treatment or had finished treatment were contacted by phone asked for participation. FGDs with visitors at TB screening camps were coordinated together with the municipal organizers. Visitors waiting for testing were approached in person and asked for participation on the same day as the FGD. All potential participants who were approached were informed about the purpose of the study and that participation was voluntary. Participants in the TB patient group and community mobiliser group with long travel distance to the location of FGD were offered economic compensation (around 5 USD) to cover travel costs. The study was presented and discussed with local community representatives. Inclusion criteria for participants were being ≥ 18 years old to legally provide consent, and for medical doctors, experience in TB care. Inclusion criteria were chosen to ensure relevant perspectives on TB care barriers and facilitators and to ensure compliance with ethical standards.

### Data collection

The data collected were qualitative, consisting of participants’ verbal responses during FGDs.

Before the start of the FGD all potential participants were informed verbally in layman’s language about the researchers, the study’s objective, possible risks to the participant, that all information shared during the discussion should be kept confidential, and that any information used in the research would be anonymized. Further, participants were informed that they were free to choose not to participate, or to opt out at any point without the need to state a reason. A sheet of written information was handed out before the FGD with time to read and ask questions about the study. Participants who could not read or write were informed of the meaning of the information sheet by a research assistant speaking local dialect. After verbal and written information was given, those who wanted to participate documented their consent by signature or fingerprint. Each FGD included a moderator, an observer and 2–5 participants. The moderator was a research assistant from Bajhang fluent in English, Nepali and the local dialect. FGDs were conducted in Nepali at health centers. The duration of FGDs were 30–75 min. The discussions followed an interview guide that was designed based on a conceptual framework of healthcare access as proposed by Levesque [22], which considers different dimensions of access, including approachability, acceptability, availability, affordability, and appropriateness. This conceptual framework was chosen to ensure an in-depth exploration of TB care barriers and facilitators. To increase reliability and validity, the interview guide was pretested in a pilot group of visitors to a TB screening camp, which resulted in minor adjustments to improve the flow of discussion. Considering the sensitive nature of TB, FGDs were conducted in secluded and undisturbed areas of the health facilities. Before the start of FGDs participants presented themselves to each other and were offered light snacks to make participants feel comfortable sharing their thoughts openly. The moderator initiated the discussion using the interview guide, while the observer took notes and suggested follow-up questions when needed to meet the study objectives. All sessions were audio-recorded, translated and transcribed into English.

### Analysis

To summarize the information collected from the FGDs, a deductive thematic analysis was conducted, building on previous knowledge of TB care access [23]. A predeveloped coding frame based on previously found barriers and facilitators in TB care was used, covering aspects of TB awareness, social and cultural factors, economic aspects, geographical aspects and aspects related to TB treatment and care providers. The coding frame was unconstrained, meaning that new categories and subcategories were made when needed [23]. A subgroup analysis was conducted for the groups of patients, TB camp visitors, medical doctors and FCHVs. The analysis of data was managed using QSR NVivo 14. All collected data was anonymized by detaching participants’ quotes from personal data before input in NVivo. No personal data was shared within the research team. The researcher reviewed the transcripts and created descriptive codes for the different values expressed by the participants. A combination of explicit and implicit codes was used [24]. Barriers were defined as factors hindering or delaying TB diagnosis and treatment. Facilitators were defined as factors promoting diagnosis and treatment. An expressed lack of a facilitator was coded as a barrier. Codes were further grouped into subcategories, categories and themes based on semantic content [24]. The analysis was dynamic, as codes and groupings of codes were continually refined throughout the process to best represent the data.

## Results

Fifty-six codes were formed, 35 of which were barriers and 21 of which were facilitators. These were grouped into 22 subcategories, five categories and three themes. The results will here be presented in a shortened version by three themes, comparing perceptions between care providers (community mobilizers (CMs) and medical doctors (MDs)) and service users (TB patients and TB camp visitors).

### Theme: individual and local community

#### Category: sociocultural influences on care-seeking behavior

Care providers and service users both generally perceived that the path from noticing initial TB symptoms to seeking care commonly takes longer than what is ideal. Different causes to delayed care seeking were raised, such as low TB awareness, stigma and traditional beliefs about TB in the community, long travel distance to health facilities, high costs and alternative medicine.

Most of the patient group described that after noticing the first symptoms they had initially resorted to common medications like paracetamol, traditional medical practices or nothing before seeking care at local health facilities. Both service users and care givers described a tendency of people to wait seeking care until or unless there was a self-perceived seriousness of disease that urgently needs medical care. Such symptoms that patients perceived as more serious were coughing with blood, pain, recurring fever and weight loss. Some patients reported initially hiding symptoms and not talking to others about it. Many patients reported that once finally reaching out to seek care at local health facilities, being referred to provincial or higher-level health facilities to get the final diagnosis could in itself cause delays.

All groups of stakeholders saw how low levels of TB awareness in the community, including limited knowledge about the cause of TB, transmission risks and TB symptoms, delay care seeking. While care providers perceived TB awareness in the community as generally low, the opinion varied among TB patients and some patients pointed out that the common knowledge is increasing. Some TB camp visitors were not familiar with TB while others were well-informed. One participant illustrated how low TB awareness contributes to continued transmission:

*“The level of awareness is very low in our part. [When] grandparents are infected with TB, they usually take care of their grandchildren, and there is a high chance of early transmission [and] relapse as an active TB.”* – MD, male

Care givers and service users agreed that information campaigns, contact tracing, volunteering programs and screening camps as effective in promoting care-seeking behavior.

Attitudes toward individuals with TB also influence care-seeking behavior. Stakeholders observed stigmatization and discrimination against people with TB, resulting in their isolation and psychosocial distress, particularly before diagnosis and treatment. Participants witnessed profound negative impacts on social life and livelihood due to TB stigma, exemplified as follows:

*“… my [relative] used to have TB … He lost his job. Even all his roommates from his village kicked him out.” –* MD, male

However, some service users also felt that the increased availability of treatment and knowledge about it had made TB easier to be open about, compared to when there was no treatment available at all:

*“At first it was very tough to talk about TB because of it being a dangerous disease just like cancer, but at this time TB has become such a common disease just like diarrhea.”- TB patient, male*

CMs particularly voiced the role of stigma and low TB awareness in delayed care seeking and perceived their role as helpful in counteracting TB stigma by providing information about TB and by showing kindness and understanding to TB-affected individuals in their communities. MDs perceived campaigning about TB through radio programs as helpful for reducing stigma.

Additionally, unequal gender dynamics were also identified as a potential barrier to seeking care, as women are generally economically reliant on husbands and have to take care of children, making it difficult to leave home for health check-ups far away, which was particularly voiced among TB camp visitors and patients living far from diagnostic services.

Furthermore, some participants, particularly TB camp visitors, expressed a lack of trust in health providers, due to initially providing the wrong diagnosis or due to shortage of medical expertise and diagnostic services like X-ray in their local health facilities. Some also expressed low trust in the political leadership to build the needed infrastructure and provide basic medical necessities as they felt their area being overlooked by the government and that the government has difficulties managing itself. Meanwhile, other expressed confidence in the care provided. These beliefs and attitudes may also affect the propensity to seek care.

### Theme: reaching care facilities

#### Category: geographical challenge of reaching TB care providers

The mobility within Bajhang is limited by its mountainous terrain, poor road conditions and remote communities. All stakeholders perceived these geographical and infrastructural constraints as major barriers for people accessing TB care. Accessing testing facilities and doctor consultations to obtain a diagnosis was perceived as particularly difficult because these services are available only in the district center, Chainpur, which may require days of traveling for people in remote areas of the district:

*“It takes days to go to the [district] center. We do not have the cost of transportation. We are old people, and Chainpur is very far for us.” –* TB camp visitor, female

In contrast, treatment facilities were perceived by both care givers and service users to be more accessible than diagnostic services because of the wide availability of DOTS clinics, although some TB patients also reported having to walk several hours to reach their DOTS clinics. The limited options of transportation to reach local health centers, in many cases only walking, are particularly challenging for elderly people and physically weaker people.

Participants recognized that access to treatment facilities has improved compared to before because services are currently reachable to some degree in the local setting, with the local health centers staffed by health assistants and support workers.

CMs viewed their roles as facilitating for people to reach care facilities by raising public awareness, investigating the situation in their communities and helping people with suspected TB reach health centers:

*“In my own village a few weeks ago, we have helped ten people to come to the local center for TB check-up, and out of these ten people, three were sent from the local center to the district center in Chainpur and diagnosed with TB, and they are receiving treatment.” –* TB volunteer, male

Caregivers perceived screening camps as facilitating the overcoming of geographic barriers, although they are very limited in number due to budget constraints. Service users also agreed with this and TB camp visitors in particular voiced the need for a more regular access to diagnostic services and doctors’ consultation at the local health posts.

##### Category: economic challenge of engaging with TB care

The economic support provided by the NTP was recognized as helpful for diagnosing and adhering to treatment. However, all categories of stakeholders described notable economic barriers remaining in the steps leading up to getting diagnosed. First, the cost of transportation to reach testing facilities; second, referrals to other care providers, often outside the district; and third, other expenses, such as lodging and food costs, including accompanying family members, for up to two weeks in Chainpur to obtain a diagnosis.

*“Medicine is for free, we don’t have to pay for it. For the transportations it almost takes 600 rupees [7 USD] so when I have to come from village up and down it almost takes 1200 rupees [14 USD]”- TB patient, male*

Furthermore, indirect costs such as becoming unemployed, whether due to stigma or weakness caused by the disease, result in a loss of income, which negatively impacts the economy of TB-affected households, which are often economically disadvantaged in Bajhang. Borrowing money was reported as a coping strategy to afford medical expenses. CMs reported lending their personal money to the person in need but were not perceived to be a sustainable strategy.

### Theme: care provider-related factors

#### Category: limited resources at local health centers

Both MDs and TB camp visitors perceived a lack of essential resources at local healthcare centers, including medical expertise and equipment. This scarcity leads to difficulties in getting diagnosed, as patients must invest time and incur travel costs to reach the district center. All groups unanimously agree that health centers must be strengthened locally with expertise and equipment so that diagnosis is possible in the local health center and not just in the district headquarters. One TB camp visitor said,

*“It would have been better service here if there was video X-ray and a doctor. If there was a video X-ray facility at the local [health center] we could do our check-up here, and in the evening, we could do our necessary work at home.” –* TB camp visitor, female

MDs highlighted the positive impact of introducing GeneXpert in the district hospital, facilitating diagnosis, identifying MDR-TB cases and reducing the need for referrals outside the district, and viewed it thereby as a strong facilitator. Some service users specifically voiced the need for better diagnostic expertise for TB affecting the glands, as it had taken them long time and many referrals to get their final diagnosis.

Care providers and service users had differing opinions regarding the DOTS scheme. Some TB patients living far from their DOTS clinics said that weekly DOTS visits were too frequent and could be a barrier to engaging with treatment. Also they felt that care providers were not open to adjust the treatment scheme to their long travels with fewer visits at the clinic. In contrast, one participant in the MD group perceived that weekly visits are too infrequent and that daily visits are preferable to facilitate compliance and completion of treatment. Service users living closer to their DOTS clinic did not perceive the same issue of weekly visits.

## Discussion

Identifying TB cases and ensuring access to TB care remain challenges in rural areas of Nepal. This study aimed to explore the barriers and facilitators to accessing TB care in Bajhang as perceived by TB patients, TB camp visitors, community mobilizers and medical doctors.

### Main findings

The main challenges to accessing TB care include low TB awareness, sociocultural stigma, geographical barriers, the economic burden of engaging with TB care and a lack of technical and professional resources at local health centers. Conversely, factors perceived to facilitate TB care access are economic support for testing and treatment, arranging TB screening camps in remote areas and community mobilizers increasing TB awareness and care engagement in villages.

These barriers are most pronounced in the early stages of healthcare—recognizing the need for care, reaching a health center, undergoing testing and receiving a diagnosis—but also in later stages, such as engaging with treatment. Early barriers prevent early treatment, which is essential for preventing advanced TB stages and transmission in the community. The early barriers may explain the relatively low number of reported TB cases in Bajhang compared to other districts, nearly half of the national average incidence [4].

### Changes needed at different levels

The findings suggest that simultaneous and integrated changes are necessary across multiple levels: individual and community, infrastructure and healthcare provider, health policy and social protection. For example, in Bajhang, recommended changes based on participants answers include strengthening social security systems for the community, improving local health centers with diagnostic equipment such as microscopy, GeneXpert, X-ray, and building a safe road network to facilitate easier mobility of people and sample transport. Addressing these challenges in isolation would be ineffective due to their interconnected nature. For example, if the government focused solely on expanding TB testing resources without tackling TB stigma, the overall impact would likely be diminished. The specifics of how interventions should be formed in a cost-effective way are outside the scope of this study. This coordinated approach is supported by the WHO multistakeholder accountability framework to end TB, which underlines the importance of multisectoral engagement [26].

### Comparison with previous studies

The results of our study align with previous research conducted in other districts of Nepal, such as Marahatta et al. and Dixit et al. [14, 15]. Both studies identified similar barriers, such as lack of health literacy, stigma and economic burden, associated with TB care. The necessity of integrated interventions, including social and economic support for patients and improved resources in local healthcare settings, was also highlighted in these studies.

Marahatta et al.’s study, which was conducted mainly in districts in mountainous regions, emphasized geographical and infrastructural barriers to reaching testing and treatment facilities. In contrast, Dixit et al.’s study, which was conducted mainly in low-elevation districts of the Terai region, revealed fewer geographical barriers but more economic and psychosocial barriers. For instance, Marahatta et al. raised the issue of inconvenient DOTS intervals for patients due to difficult mobility in the district, as in this study. Dixit et al. also identified frequent DOTS visits as challenging for the poor working population and women due to a lack of free time. This study recognized patients preferring longer intervals to make treatment more manageable and doctors preferring more frequent visits to ensure treatment adherence. This study aligns with previous research suggesting the need for individualized DOTS schemes or alternative treatment strategies other than the standard scheme, which seems to mainly fit a smaller section of the population [27].

### Can facilitators overcome barriers?

The facilitators identified in this study, including volunteer programs, FCHVs and TB volunteers, screening camps and economic support programs, were perceived as valuable throughout all stages of seeking and engaging with TB care. However, these facilitators are limited by resource constraints. For example, the TB volunteer program, currently operational in one ward of a single municipality of Bajhang, has room for expansion. Economic support covers only a fraction of TB-related costs, and nutritional support is only provided for MDR-TB patients [4]. In conclusion, while promising, these facilitators are constrained in their effectiveness and coverage of the population due to resource limitations.

### Significance

The findings of our study strengthen previous knowledge on how seeking and engaging with TB care is hindered by economic barriers in poor countries and in vulnerable subpopulations. Globally, a growing number of studies conducted in both urban and rural areas of LMICs have identified TB care access as a multidimensional challenge, including economic, infrastructural, and psychosocial aspects, and the challenges are deeply connected with broader issues such as poverty, malnourishment and HIV infection [28, 29]. While individual studies may emphasise different aspects depending on the study, they all emphasise the need for coordinated multisectoral action to effectively reduce TB [30]. This study aligns with previous research by highlighting the multidimensional challenges to TB care access within poor communities.

### Strengths and limitations

The results should be interpreted within the context in which the study was conducted. A qualitative study does not assume neutrality, and the researcher’s sociocultural background may have influenced outcomes. The use of translators leads to potential inaccuracies, addressed by back-translating selected excerpts for validation [31]. The trustworthiness could be improved by peer-reviewing transcripts and analysis by an independent researcher. Gender-separated FGDs with patients were planned but not conducted due to time constraints, which could have provided insights into the influence of gender dynamics on care access.

### Future studies

Future research should explore the potential of telemedicine in overcoming geographical barriers to TB testing and treatment, such as digital DOTS visits or the use of drone networks for the transportation of sputum samples and delivery of medication to remote areas, which has started to be explored in Nepal [32]. The feasibility and potential benefits of such strategies should be studied in Bajhang.

## Conclusions

As perceived by key stakeholders, the remaining barriers to accessing TB care in Bajhang include low levels of TB awareness, social stigma, high economic costs, and geographic inaccessibility. Facilitators for accessing TB care include economic support programs, increasing the resources of local healthcare centers, TB screening camps and volunteer programs. Priority actions should focus on the early stages of care seeking and address individual, community, infrastructural and health policy levels.

## Data Availability

No datasets were generated or analysed during the current study.
